# Mechanisms of pulmonary dysfunction after on-pump and off-pump cardiac surgery: a prospective cohort study

**DOI:** 10.1186/1749-8090-2-11

**Published:** 2007-02-14

**Authors:** AB Johan Groeneveld, Evert K Jansen, Joanne Verheij

**Affiliations:** 1 Department of Intensive Care, VU University Medical Center, Amsterdam, The Netherlands; 2 Department of Cardiothoracic Surgery VU University Medical Center, Amsterdam, The Netherlands; 3 Institute for Pathology VU University Medical Center, Amsterdam, The Netherlands; 4 Institute for Cardiovascular research, VU University Medical Center, Amsterdam, The Netherlands

## Abstract

**Background:**

Pulmonary dysfunction following cardiac surgery is believed to be caused, at least in part, by a lung vascular injury and/or atelectasis following cardiopulmonary bypass (CPB) perfusion and collapse of non-ventilated lungs.

**Methods:**

To test this hypothesis, we studied the postoperative pulmonary leak index (PLI) for ^67^Ga-transferrin and (transpulmonary) extravascular lung water (EVLW) in consecutive patients undergoing on-pump (n = 31) and off-pump (n = 8) cardiac surgery. We also studied transfusion history, radiographs, ventilatory and gas exchange variables.

**Results:**

The postoperative PLI and EVLW were elevated above normal in 42 and 29% after on-pump surgery and 63 and 37% after off-pump surgery, respectively (ns). Transfusion of red blood cell (RBC) concentrates, PLI, EVLW, occurrence of atelectasis, ventilatory variables and duration of mechanical ventilation did not differ between groups, whereas patients with atelectasis had higher venous admixture and airway pressures than patients without atelectasis (P = 0.037 and 0.049). The PLI related to number of RBC concentrates infused (P = 0.025).

**Conclusion:**

The lung vascular injury in about half of patients after cardiac surgery is not caused by CPB perfusion but by trauma necessitating RBC transfusion, so that off-pump surgery may not afford a benefit in this respect. However, atelectasis rather than lung vascular injury is a major determinant of postoperative pulmonary dysfunction, irrespective of CPB perfusion.

## Background

Cardiac surgery involving cardiopulmonary bypass (CPB) can be complicated by pulmonary dysfunction after surgery, sometimes necessitating prolonged mechanical ventilation, the causes of which remain largely unclear [1,2]. A lung vascular injury occurs in 30–50% of patients, as measured by a postoperative rise in pulmonary capillary permeability by the ^67^Ga-transferrin pulmonary leak index (PLI) related to the duration of CPB [3,4]. The injury is thought to originate from proinflammatory responses, among others, and to contribute to postoperative oedema, i.e. a rise in extravascular lung water (EVLW), with (transient) gas exchange and mechanical abnormalities of the lungs [2-11]. Other factors that might affect lung vascular injury include surgical trauma [9,10], and the use of blood products increasing the risk for transfusion-related lung injury (TRALI) [12,13]. In fact, the necessity to transfuse is associated with increased morbidity and mortality [12,14]. During CPB, the lungs are not ventilated, promoting atelectasis and the latter, albeit hard to recognize [15], may further contribute to postoperative pulmonary dysfunction and predispose to infection [4]. Moreover, atelectasis may already develop prior to surgery at the induction of anaesthesia [15]. Even though biocompatibility of CPB systems have improved over decades, off-pump bypass surgery has been revitalized, potentially offering less pulmonary complications as compared to on-pump surgery [16], although only few [11,17-21] of the prospective randomised clinical trials with primary or secondary pulmonary endpoints [11,17-27], including gas exchange, duration of intubation and mechanical ventilation and occurrence of pneumonia favored off-pump surgery. Non-randomized studies indicate similar [5,28,29] or better [6,23,30-32] postoperative radiography, lung compliance and gas exchange and less pulmonary complications with off-pump surgery. The mechanisms remained unclear, however, and might relate to prevention of a proinflammatory responses, lung vascular injury and atelectasis, by obviating CPB and collapse of non-ventilated lungs [7,8,30,32].

Taken together, the mechanisms of postoperative pulmonary dysfunction in on- and off-pump cardiac surgery remain unclear, while having therapeutic consequences [2,4,7,30,33]. To test the hypothesis that lung vascular injury and atelectasis are not related to CPB per se, we studied consecutive patients undergoing on- and off-pump surgery with respect to transfusion history, PLI, EVLW (transpulmonary dilution), and ventilatory and radiographic variables, within 3 h after admission to the intensive care unit (ICU).

## Patients and methods

This a prospective observational cohort study, approved by the Ethical Committee of the VU University Medical Center, including 39 consecutive patients after elective cardiac surgery involving CPB or off-pump surgery. In this university hospital, about 650 open heart procedures are performed per year, of which about 20% without CPB. Written informed consent was obtained pre-operatively in eligible patients (n = 52). The inclusion criteria were the presence of a pulmonary artery (n = 36) or central venous catheter (n = 3), postoperatively. Exclusion criteria were an age above 79 years, a life expectancy less than 24 h and signs of cardiac failure or overhydration, defined by a central venous pressure (CVP) above 13 or pulmonary capillary wedge pressure (PCWP) above 15 mm Hg, at arrival in the ICU. On the day of the surgery, anaesthesia was induced and 50–100 mg of dexamethasone was administered. Catheters were inserted for haemodynamic measurements and blood sampling. After tracheal intubation, the lungs were volume-controlled ventilated with a tidal volume (V_t_) of 8–10 mL·kg^-1 ^resulting in an end-tidal CO_2 _concentration between 4 and 5%, using an O_2_-air mixture with an inspiratory O_2 _fraction (F_I_O_2_) of 40% and a positive end-expiratory pressure (PEEP) of 5 cm H_2_O (I:E 1:2). We do not routinely use ventilatory recruitment maneuvers. The patients underwent CPB during which the lungs were not ventilated. The system was primed with Ringer's lactate and gelatin 4%. After heparinization (300 IU/kg), CPB (Stockert-Sorin S3, Sorin Biomedica, Mirandola, Modena, Italy) was started. Non-pulsatile flow rate was maintained at 2–3 L·min^-1^·m^-2^. Patients were cooled to 32°C nasopharyngeal temperature. Mean arterial pressure (MAP) was maintained at 50–80 mm Hg during CPB and if the MAP declined to less than 50 mm Hg, the blood flow rate was increased or vaso-active drugs were given. After aortic cross-clamping, all patients received hyperkalaemic cardioplegia for myocardial protection (4°C). Patients were weaned from CPB using inotropic support, if necessary. Off-pump surgery was performed with two deep pericardial stitches and a mechanical stabilizer (Guidant, Acrobat mechanical stabilizer, Santa Clara, USA). Total arterial revascularization with both mammary arteries was attempted. The left and right pleura were opened routinely for mobilization of the arteries. Fluids and inotropic drugs were given to keep the venous oxygen saturation above 0.70, as monitored by a continuous cardiac output catheter (Vigilance monitor, Edwards Lifesciences, USA). After termination of procedures, heparin was neutralized. Autologous blood and residual volume from the extracorporeal circuit were infused as first-choice fluid administration. Guided by low systemic and filling pressures, saline, gelatin or starches were infused. If the haemoglobin concentration was less than 6 mmol/L, leukoreduced red blood cell concentrates were infused. At the end of surgery, a 4F introducing sheath (Arrow, Reading, USA) was inserted into the femoral artery, for use in the study protocol.

The EVLW was measured with help of the thermal-dye technique [4,34]. A 3F fiberoptic thermodilution catheter was inserted in the femoral artery sheath. Fifteen mL of ice cold indocyanine green (ICG), 1 mg·mL^-1 ^D5 W, was injected in a central vein and the thermal-dye dilution curve obtained in the femoral artery (COLD Z-021, Pulsion Medical Systems, Muenchen, Germany). This allowed calculation of the transpulmonary cardiac output and the EVLW (normal <7 mL/kg) [34]. EVLW is typically two to three-fold elevated in case of overt (radiographic) pulmonary oedema [34]. Measurements were done in duplicate and averaged. The cardiac output was indexed to body surface area (cardiac index, CI). Transpulmonary dilution indicators of preload included the global end-diastolic volume index (GEDVI, n 700–900 mL·m^-2^) and the intrathoracic blood volume index (ITBVI, n 900–1200 mL·m^-2^).

The PLI was measured as described previously [3,4,35]. In brief, autologous red blood cells were labelled with ^99 m^Tc (11 MBq, physical half-live 6 h; Mallinckrodt Diagnostica, Petten, The Netherlands). Transferrin was labeled in vivo, following i.v. injection of ^67^Ga-citrate, 4.5 MBq (physical half-live 78 h; Mallinckrodt Diagnostica, Petten, The Netherlands). Patients were in the supine position and two scintillation detection probes (Eurorad C.T.T., Strasburg, France) were positioned over the lung apices. Starting at the time of injection of ^67^Ga, radioactivity was detected every minute, during 30 minutes. The count rates were corrected for background radioactivity, physical half-life and spill-over and expressed as counts per minute (CPM) per lung field. Until 30 minutes after ^67^Ga injection, blood samples were taken. Each blood sample was weighed and radioactivity was determined with a single well well-counter, corrected for background, spillover and decay (LKB Wallac 1480 WIZARD, Perkin Elmer, Life Science, Zaventem, Belgium). Results were expressed as CPM·g^-1^. For each blood sample, a time-matched CPM over each lung was taken. A radioactivity ratio was calculated, (^67^Ga-lung/^99 m^Tc-lung)/(^67^Ga-blood/^99 m^Tc-blood), and plotted against time. The PLI was calculated, using linear regression analysis, from the slope of increase of the radioactivity ratio divided by the intercept. The PLI represents the transport rate of ^67^Ga from the intravascular to the extravascular space of the lungs and is therefore a measure of pulmonary vascular permeability [3,4,35]. The values for both lung fields were averaged. The upper limit of normal for the PLI is 14.1 × 10^-3^/min, as obtained in preoperative vascular and cardiac surgery patients and the measurement error is about 10% [3,35]. The PLI is typically elevated more than three- to fourfold in ARDS [3,35].

### Radiography and lung injury score (LIS)

The latter was calculated from the number of quadrants on the chest radiograph with opacities, the PEEP level, the arterial PO_2 _(P_a_O_2_)/inspiratory O_2 _fraction (F_i_O_2_) and the total respiratory dynamic compliance [36]. The compliance was calculated from tidal volume/(plateau pressure-PEEP), mL/cm H_2_O. The chest radiograph was scored by a consultant radiologist, blinded to the study, who evaluated the number of quadrants with alveolar opacities, ranging from 0 to 4. In addition, the presence of blurring of the left hemidiaphragm and costophrenic angle by alveolar opacification was recorded as radiographic evidence for left lower lobe atelectasis [32]. The LIS ranges between 0 (no injury) to 4, with values above 2.5 indicative of ARDS, and between 0 and 2.5 of ALI [36].

### Protocol

After surgery, the patients were admitted to the ICU. The patient was connected to the ventilator (Evita 4, Dräger, Lübeck, Germany) and volume-controlled ventilated with similar settings as during surgery. Active external rewarming was started, when needed. Demographics were recorded, including the medical history, preoperative Euroscore [37] and left ventricular ejection fraction, transfusion history during surgery and the Acute Physiology and Chronic Health Evaluation (APACHE-II) score upon ICU admission. Measurements of EVLW, ^67^Ga-transferrin PLI and haemodynamics were performed, and an anteroposterior chest radiograph was made, within 3 h after ICU admission. Haemodynamic variables were measured after calibration and zeroing to atmospheric pressure at mid-chest level (Tramscope^R^, Marquette, Wisc., USA). Mean pulmonary artery pressure (MPAP), CVP and, after balloon inflation, the PCWP were taken at end-expiration, with patients in the supine position, if adequate tracings for the latter could be obtained (n = 24). Arterial blood samples were obtained for determinations of lactate, partial O_2_/CO_2 _pressures and O_2 _saturations (Rapidlab 865, Bayer Diagnostics, Tarrytown, NY, USA, at 37°C), and haemoglobin/haematocrit (Roche/Hitachi 747, Roche Diagnostics Corporation, Indianapolis, IN, USA). Mixed (n = 36) or central (n = 3) venous blood was taken simultaneously for measurement of partial pressures and saturations. Venous admixture (Q_s_/Q_t_), oxygen delivery (DO_2_) and O_2 _consumption (VO_2_) were calculated according to standard formulae. The plasma colloid osmotic pressure (COP) was measured by a membrane osmometer (Osmomat 050, Gonotex, Berlin, molecular cut-off at 20 kDa, normal about 24 mm Hg). The F_I_O_2_, tidal volume, plateau inspiratory pressure and PEEP (cm H_2_O) were taken from the ventilator. Doses of vasoactive drugs were recorded. Patients were taken care of by intensive care physicians not involved in the study and followed until extubation and discharge/death in the ICU. The duration of mechanical ventilation was defined as the interval from admission to extubation.

### Statistical analysis

Data were summarized as median and range and groups were compared with help of the non-parametric Mann-Whitney U test for unpaired data. Fisher's exact test was used to compare frequencies and the Pearson correlation coefficient to express relations. Exact P values < 0.05 are given unless < 0.0001. We post hoc calculated the number of patients needed to reach statistical significance (at a power of 80%) for the observed difference in PLI and atelectasis between groups, which otherwise contrasted with the a priori hypothesis, and these amounted to 90 and 320 per group, respectively.

## Results

Table 1 describes patients characteristics. All but one patient survived to hospital discharge. Only on-pump patients underwent aortic valve replacements in addition to coronary artery surgery. The lowest temperature during surgery and the number of pleurotomies differed between groups. Table 2 describes that on-pump and off-pump cardiac surgery patients differed in temperature, CI, DO_2 _and S_v_O_2 _at the time of study in the ICU. Lung compliance and oxygenation were somewhat abnormal after surgery but similar among groups. There was no intergroup difference in number of RBC concentrates transfused, PLI, EVLW, occurrence of atelectasis and duration of mechanical ventilation. In coronary artery patients only, the PLI after on-pump surgery (n = 20) was 18 (7–73) and after off-pump 18 (13–27) × 10^-3^/min (n = 7; ns), while CI was still lower in the former (P = 0.022). In on-pump and off-pump patients together, but not in each group separately, radiographic evidence for atelectasis of the left lower lung lobe was associated with higher venous admixture (median difference 4.4%, P = 0.037), P_plat _(median difference 2.7 cm H_2_O, P = 0.049) and LIS (1.1 [0.5–2.7] vs 0.7 [0.2–1.2], P = 0.030) than the absence of atelectasis, while PLI and EVLW did not differ. In contrast, the PLI directly related to the number of RBC units transfused (r = 0.37, P = 0.025, in on-pump and off-pump surgery patients), and not to the duration of CPB. Chronic obstructive pulmonary disease was neither associated with an elevated PLI.

**Table 1:** Patient characteristics

	Off pump n = 8	On pump n = 31
**Prior to and during surgery**		
Age, year	65 (56–74)	62 (38–75)
Sex, m/f	8 (100)	23 (74)/8 (26)
Euroscore	2 (0–5)	3 (0–7)
LV ejection fraction <50%	1 (12)	6 (19)
COPD	0	5 (16)
CPB time, min	0	114 (40–198)
Aortic clamp time, min	0	76 (26–155)
Pleurotomy	8 (100)	19 (61)*
Aortic valve replacement	0	9 (19)
Closure atrial septal defect	0	2 (6)
Number of grafts	4 (2–5)	4 (1–7)
Lowest temperature, °C	36.0 (35.7–36.2)	31.4 (27.8–35.8)*
Red blood cell concentrates	0 (0–4)	0 (0–5)
**In the ICU**		
APACHE II	8 (3–13)	9 (2–15)
Dopamine, mg/h	8 (0–28)	8 (0–40)
Nitroglycerin, mg/h	0.5 (0–1)	0.5 (0–2.5)

Median (range) or number of patients, where appropriate. Abbreviations: ICU, intensive care unit; LV left ventricle; COPD, chronic obstructive pulmonary disease; CPB, cardiopulmonary bypass; APACHE II, acute physiology and chronic health evaluation. *P = 0.042; *P < 0.0001

**Table 2:** Off pump versus on pump cardiac surgery

	Off-pump n = 8	On-pump n = 31	P
**Haemodynamic/metabolic**			
Temperature, °C	36.5 (35.6–37.2)	35.7 (34.5–36.5)	0.008
HR,·min^-1^	74 (57–87)	72 (55–101)	
MAP, mm Hg	70 (57–104)	75 (52–104)	
CVP, mm Hg	3 (1–7)	5 (0–12)	
PCWP, mm Hg	6 (5–7)	7 (1–13)	
MPAP, mm Hg	13 (11–28)	15 (8–23)	
CI, L·min^-1^·m^-2^	3.9 (2.7–4.8)	2.8 (2.1–4.3)	0.008
GEDVI, mL·m^-2^	970 (620–1131)	783 (537–1180)	
ITBVI, mL·m^-2^	1180 (767–1342)	1019 (641–1498)	
DO_2_, L·min^-1^·m^-2^	503 (385–576)	391 (229–546)	0.008
VO_2_, L·min^-1^·m^-2^	117 (78–192)	120 (26–189)	
Lactate, mmol/L	0.9 (0.6–1.2)	1.4 (0.7–4.1)	<0.0001
COP, mm Hg	19 (16–25)	19 (15–24)	
Haemoglobin, mmol/L	5.5 (4.9–7.1)	5.9 (4.3–7.4)	
Haematocrit	0.25 (0.23–0.32)	0.28 (0.20–0.33)	
			
**Pulmonary**			
PLI, × 10^-3^·min^-1^	18 (13–27)	14 (6–73)	
Supranormal PLI	5 (63)	13 (42)	
EVLW, mL·kg^-1^	6.7 (4.8–9.7)	5.9 (2.1–20.0)	
Supranormal EVLW	3 (37)	9 (29)	
V_t_, mL	580 (400–660)	550 (395–1110)	
P_plat_, cm H_2_O	17(14–19)	17 (13–33)	
PEEP, cm H_2_O	6 (5–10)	7 (4–16)	
Compliance_tot,respir,dyn_, cm H_2_O·mL^-1^	64 (40–83)	50 (37–91)	
P_a_O_2_, mm Hg	128 (98–176)	119 (84–189)	
P_a_O_2_/F_I_O_2_	299 (251–463)	280 (140–485)	
S_a_O_2_	0.98 (0.98–0.99)	0.98 (0.96–0.99)	
S_v_O_2_	0.77 (0.69–0.81)	0.73 (0.61–0.95)	0.030
Venous admixture, %	17 (9–21)	16 (5–62)	
Quadrants on X-ray with densities	0 (0–1)	0 (0–3)	
Atelectasis	5 (63)	16 (52)	
Lung injury score	0.7 (0.2–1.2)	1.0 (0.2–2.7)	
Duration MV, h	11 (6–23)	11 (5–494)	

Median (range) or number of patients where appropriate. Abbreviations: HR, heart rate; MAP, mean arterial pressure; CVP, central venous pressure; PCWP, pulmonary capillary wedge pressure; MPAP, mean pulmonary artery pressure; CI, cardiac index; DO_2_, oxygen delivery; VO_2_, oxygen consumption; COP, colloid osmotic pressure; PLI, pulmonary leak index; EVLW, extravascular lung water; V_t_, tidal volume; P_plat_, plateau pressure; PEEP, positive end-expiratory pressure; F_I_O_2_, inspiratory O_2 _fraction; P/SO_2_, O_2 _partial pressure/saturation; a, arterial; v, mixed venous; MV, mechanical ventilation.

## Discussion

Our study suggests that the mechanisms and their relative importance in causing pulmonary dysfunction after cardiac surgery do not depend on CPB perfusion.

The frequency and magnitude of postoperative PLI and EVLW elevations conform with previous studies on cardiac surgery [3,4,6,7]. Indeed, a lung vascular injury and oedema may be associated with deteriorated gas exchange and pulmonary compliance after surgery, although this is controversial [2,3,5-7]. We do not know whether the transfusion of RBC concentrates was a marker or a mediator of severe lung vascular injury and a high PLI, as in TRALI [12,13]. Extensive trauma and release of proinflammatory mediators affecting the lungs may have been the underlying common factor, although a direct contribution of infused RBC concentrates, as in TRALI, cannot be excluded [8-10,12,13]. We did not observe a difference in transfusion requirements until inclusion in the study between on-pump and off-pump surgery as observed by some investigators [8,25,27], even though superiority of the latter technique in this respect has been suggested [9,14,16-19,21,24,31,32]. Hence, the underlying trauma rather than amelioration of CPB-related proinflammatory responses [8-10] may have caused the lung vascular injury, similarly in on-pump and off-pump patients. However, atelectasis superimposed on the lung vascular injury was a major determinant of oxygenation impairment and airway pressure requirements in our patients, without affecting dynamic compliance and irrespective of CPB perfusion. That atelectasis is of greater importance for postoperative lung gas exchange and ventilatory abnormalities than increased permeability oedema has been suggested before [4]. The data agree with rapidly developing atelectasis during induction of anaesthesia, prior to surgery [15], and may also explain the positive effect of continuation of airway pressure or ventilation during surgery on postoperative lung function, observed in previous studies [30,33].

Our data do not suggest any amelioration of lung vascular injury and atelectasis by off-pump as compared to on-pump surgery, and similar degree of pulmonary dysfunction and duration of mechanical ventilation in the ICU, as observed before, even though mechanisms in these studies were not addressed [8,20-23,25-29]. In other observational studies and randomized trials [2,11,17-21,30,31], however, authors found off-pump versus on-pump coronary artery surgery to be associated with less pulmonary complications, less oxygenation impairment, earlier extubation, shorter mechanical ventilation and less often pneumonia, but these observations also remained largely unexplained. In contrast, other authors reported no differences in lung mechanical changes, but this is controversial too [5,6,11,23,29,30]. Our results finally do not agree with the suggestion that off-pump surgery is associated with less radiographic atelectasis than on-pump surgery [32], which, otherwise, was not confirmed in other studies [11,29]. In all three studies [11,29,32], however, the occurrence of radiographic alveolar oedema was rare, as in our study, and did not differ among groups.

On-pump and off-pump cardiac surgery patients only differed in temperature, cardiac output and tissue oxygenation after cardiac surgery, whereas preoperative cardiac function did not differ between groups. The difference in cardiac output and oxygen delivery is unlikely explained by a difference in body temperature, when, at the time of study, rewarming of patients undergoing hypothermic CPB was not yet completed. The VO_2 _was similar among groups, in accordance with the literature (39), so that S_v_O_2 _was lower and O_2 _delivery higher after off-pump surgery. Together with similar filling pressures, the data suggest a diminished cardiac function and tissue oxygenation after on-pump as compared to off-pump surgery, as observed before, and possibly related to greater myocardial damage during hypothermic cardioplegia [17,19,23,24,26,27,31]. Together with high lactate levels in the CPB group, the similar VO_2 _among groups, suggests greater and partially unmet tissue O_2 _demands after hypothermic CPB perfusion than off-pump surgery, even though body temperature was still lower in the former during rewarming.

Limitations of the study include a limited comparability of groups and number of non-randomized patients. We wanted to study the mechanisms of postoperative pulmonary dysfunction in patients undergoing the two techniques rather than comparing them in outcomes. Although the number of study patients was large enough to detect a difference in cardiac function, we cannot exclude that the number was too small to demonstrate a small difference in pulmonary function among techniques. However, off-pump surgery patients tended to have more often elevated PLI, EVLW and atelectasis than on-pump patients, postoperatively, in contrast to expectations, so that a difference disfavoring on-pump surgery may not have been missed and the conclusion that the alterations are not caused by CPB is still valid. In any case, we post hoc calculated that a considerable number of patients would have been necessary to include, to reach a statistically significant difference (at a power of 80%) favoring on pump surgery regarding the occurrence of an elevated PLI and atelectasis after surgery. From our data, we cannot judge the contribution of pleurotomy and surgery on internal mammary arteries on the PLI and atelectasis after off-pump surgery, although no influences on postoperative pulmonary dysfunction have been suggested before [28].

## Conclusion

The occurrence of lung vascular injury inferred from an elevated PLI and of atelectasis in about half of patients after cardiac surgery are not caused by CPB. The major factor causing postoperative pulmonary dysfunction is atelectasis rather than lung vascular injury, regardless of CPB perfusion and favoring the open lung concept for mechanical ventilation starting at the time of surgery [30,33].
